# Protection is not always a good thing: The immune system’s impact on gene therapy

**DOI:** 10.1590/1678-4685-GMB-2022-0046

**Published:** 2022-07-15

**Authors:** Martiela Vaz de Freitas, Lariane Frâncio, Laura Haleva, Ursula da Silveira Matte

**Affiliations:** 1 Hospital de Clínicas de Porto Alegre, Centro de Pesquisa Experimental, Laboratório Células Tecidos e Genes, Porto Alegre, RS, Brazil.; 2 Universidade Federal do Rio Grande do Sul, Programa de Pós-Graduação em Genética e Biologia Molecular, Porto Alegre, RS, Brazil.; 3 Universidade Federal do Rio Grande do Sul, Instituto de Biociências, Porto Alegre, RS, Brazil.; 4 Hospital de Clínicas de Porto Alegre, Núcleo de Bioinformática Centro de Pesquisa Experimental, Porto Alegre, RS, Brazil.; 5 Universidade Federal do Rio Grande do Sul, Departamento de Genética, Porto Alegre, RS, Brazil.

**Keywords:** Gene delivery, gene therapy, immune response

## Abstract

There are many clinical trials underway for the development of gene therapies, and some have resulted in gene therapy products being commercially approved already. Significant progress was made to develop safer and more effective strategies to deliver and regulate genetic products. An unsolved aspect is the immune system, which can affect the efficiency of gene therapy in different ways. Here we present an overview of approved gene therapy products and the immune response elicited by gene delivery systems. These include responses against the vector or its content after delivery and against the product of the corrected gene. Strategies to overcome the hurdles include hiding the vector or/and the transgene product from the immune system and hiding the immune system from the vector/transgene product. Combining different strategies, such as patient screening and intelligent vector design, gene therapy is set to make a difference in the life of patients with severe genetic diseases.

## Gene therapy

According to the MedlinePlus (2019) definition, gene therapy is a treatment that involves introducing genetic material into cells to compensate for the function of abnormal genes or to produce a therapeutic protein. Therapeutic gene therapy is performed only in somatic cells, as germline gene therapy is not approved. In addition, all protocols must assure that the transgene will not pass onto the patient’s offspring. The delivery of the therapeutic genetic material can be performed *ex vivo* by systemic delivery or not (also called *in situ*) or *in vivo*.

In *ex vivo* therapies, the cells to be corrected are harvested, the gene transfer is performed in the laboratory, and cells are reinfused into the patient after correction. It is the preferred method for targeting bone marrow-derived cells in cancer treatment, including CAR-T cells (He et al., 2020; Sterner and Sterner, 2021).

Of course, this is not an option for many target cells. In such cases, *in vivo* or *in situ* methods must be used. The first is the systemic administration of a vector carrying the therapeutic genetic material. And is used for systemic genetic diseases (Soofiyani et al., 2013), and the therapeutic genetic material tends to be uptaken by the liver (Jacobs et al., 2012). In contrast, the latter is a particular type in which the therapy administration occurs directly into the target cells or tissue. It is a method used to treat some types of cancer, such as melanoma or brain tumors, and genetic diseases like muscular dystrophy (Hromic-Jahjefendic and Lundstrom, 2020; Banerjee et al., 2021; Elangkovan and Dickson, 2021).

**Figure 1 -:**
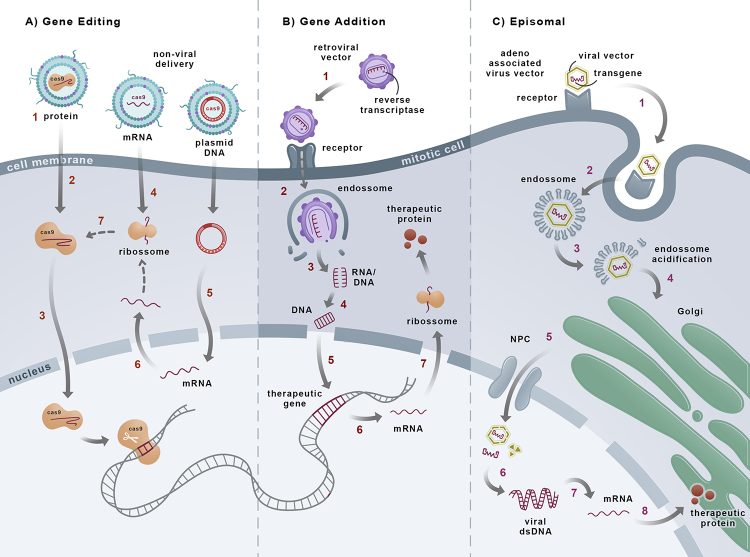
Gene therapy strategies. **A**) Gene editing: CRISPR/Cas9 may be delivered as plasmid DNA, mRNA, or protein (1). In this example, the non-viral vector binds to the cellular membrane. After endocytosis into the cell, the particles escape from the endo/lysosome (2). Protein delivery is instantaneous and transient, results in the most immediate onset of gene editing, and avoids the concern of permanently integrating CRISPR genes into the host genome (3). Transferred mRNA must be released into the cytosol to enable mRNA translation to protein (4). Plasmid DNA needs to be translocated into the nucleus. The target cell’s native transcription mechanism must be recruited to transcribe the gene into mRNA (5) and transport the mRNA into the cytoplasm (6). There, it will be translated into the protein, which must be transported back into the nucleus and modify the cell DNA (7). **B**) Gene addition: 1. In this example, the retroviral vector binds to the receptors on the cell surface and enters the cytoplasm through endocytosis (2). Once the endosome releases its content (3), the ssDNA is converted to dsDNA (4). It then gains access to the cell nucleus once the cell is in mitosis (5). The gene is inserted into the host cell DNA and transcribed into mRNA (6). In the cytoplasm, the ribosome translates it to the therapeutic protein (7). **C**) Episomal: In this example, the adeno-associated viral vector binds to receptors at the cell surface (1), and endocytosis occurs (2). The acidification process inside the endosome leads to vector liberation (3), and the Golgi-mediated capsid transport begins (4). After, the viral vector enters the nucleus through the nuclear pore complex (NPC) (5). The ssDNA is released from the vector and converted into dsDNA (6). Then, the episomal foreign DNA is transcripted into mRNA (7) and translated into the therapeutic protein (8).

In addition to traditional gene therapy, gene editing is a novel tool for correcting gene-caused diseases. Gene editing relies on three main strategies, as shown in Figure 1,that aim to modify the cell’s DNA, promoting a local and definitive correction of the targeted cells. A similar approach, gene addition, seeks to insert an extra sequence coding for the desired proteins on the cell’s DNA. 

**Figure 2 -:**
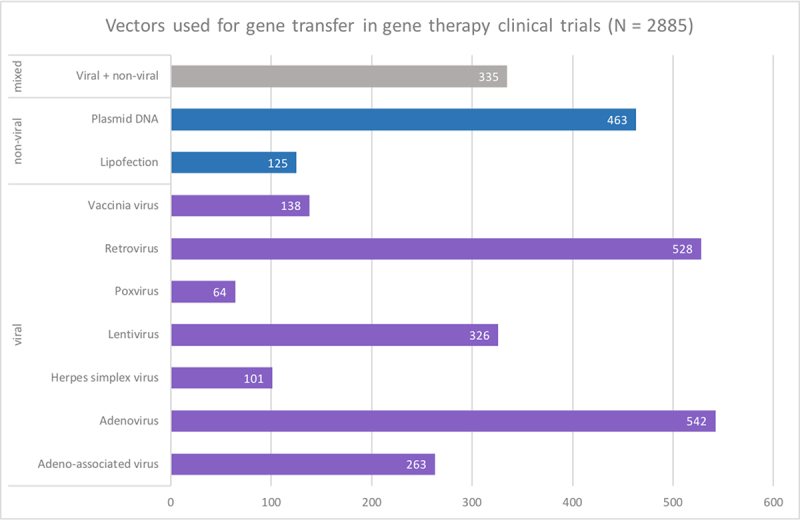
Types of vectors used in clinical trials, according to (Ginn et al., 2018).

All methods described above require strategies to transfer the genetic material to target cells. Although many pre-clinical protocols use non-viral methods such as naked DNA, liposomes, or nanoemulsions (Ramamoorth and Narvekar, 2015), most clinical trials rely on viral vectors taking advantage of viruses’ natural ability to use the host’s cell machinery to express their genetic content. Figure 2 shows the main types of viral vectors used in clinical trials, whereas Table 1 presents their characteristics.

**Table 1 -:** Characteristics of the leading viral vectors used in clinical trials.

Vector	Strategy	Packing Capacity	Infection	Integration	Tranduction Efficiency	Expression	Immunogenic potential
Adenovirus	*in vivo*	8 kb	Dividing and non-dividing cells	Episomal	High	Transient	High
Retrovirus	*ex vivo*	8 kb	Dividing cells	Integrative	Moderate	Stable	Moderate
Lentivirus	*ex vivo*	8 kb	Dividing and non-dividing cells	Integrative	Moderate	Stable	Moderate
Adeno-associated virus	*in vivo*	4.5 kb	Dividing and non-dividing cells	Episomal	Moderate	Transient/Stable	Low

**Figure 3 -:**
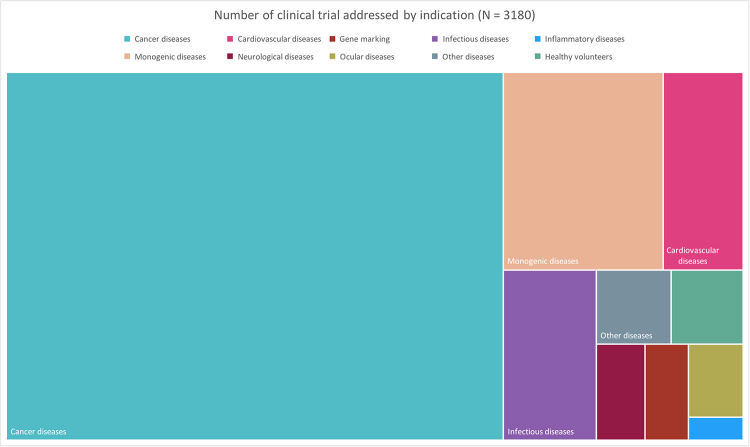
Application of gene therapy in clinical trials according to Wiley Gene Therapy Clinical Trial Databases (Ginn et al., 2018). Almost 70% of all clinical trials are designed for cancer diseases (light green), while monogenic diseases (coral) account for approximately 11%.

Gene therapy has gained momentum for treating different diseases worldwide with several clinical trials underway (Figure 3) and has made its way to the market with over ten approved products (Table 2). However, it is interesting to note that even though the definition of gene therapy refers to “genetic material”, RNA-based therapies (Table 3) are not considered gene therapy products by most regulatory agencies, including the FDA (USA), EMA (Europe) and ANVISA (Brazil). Several RNA-based products have been commercially approved worldwide. It is important to note that RNA-based therapies refer to products directed to decrease or abolish protein translation and consist of oligonucleotides that interact with mRNA. On the other hand, “traditional gene therapy” aims to provide de novo protein synthesis, thus leading to gene addition or replacement.

**Table 2-:** Approved gene therapies for gene addition or gene replacement.

Type	Name	Indication	Method	Immune-related issues*	Situation
*in vivo*
	Gendicine	Head and neck squamous cell carcinoma	Recombinant human serotype-5 adenovirus	Fever, chill, pain at the injection site, fatigue, nausea, and diarrhea (Zhang et al., 2018).	2003 - Approved
	Oncorine	Nasopharyngeal carcinoma	Recombinant human Adenovirus type 5 injection, H101	Fever, local pain at the injection site, and flu-like symptoms. (Ma *et al.,* 2009).	2005 - Approved
	Rexin-G	Metastatic pancreatic cancer	Retroviral vector	Chill, fatigue and headache. No serious drug-related adverse events (AE) were reported. (Chawla et al., 2010)	2007 - Approved
	Neovasculgen	Atherosclerotic Peripheral Arterial Disease (PAD)	Plasmid DNA	Prophylactic treatment with acetylsalicylic acid to decrease risk of cardiovascular ischemic event. No AEs related. (Deev et al., 2015)	2010 - Approved
	Glybera	Lipoprotein Lipase Deficiency (LPLD)	Adeno-associated virus serotype I	Immune response against AAV even with use of immunosuppressants.	2012 - Approved 2014 - Withdrawn
	Imlygic	Melanoma	Genetically manipulated oncolytic herpes simplex virus type 1 (HSV)	Flu-like illness, fevers and chills. Muscle pain (myalgia), painful/swollen joints (arthralgia), limb pain, vasculitis and glomerulonephritis (very rare). Autoimmmune reactions. Plasmocytoma.	2015 - Approved
	Luxturna	Inherited retinal dystrophies	Adeno-associated virus	Acute serious liver injury, acute liver failure, and elevated aminotransferases.	2017 - Approved
	Zolgensma	Treatment of Spinal Muscular Atrophy (Type I)	Non-replicating recombinant AAV9	Acute and chronic GvHD, febrile neutropenia, haemoglobin decreased, platelet count decreased.	2019 - Approved
	Delytact	Residual or recurrent glioblastoma	Oncolytic virus therapy - replication-conditional Herpes simplex virus type 1	Pyrexia, brain oedema, cytopenia, seizure, haemorrhage, infection, normal pressure hydrocephalus, and autoimmune diseases involving the central nervous system.	2021 - Conditional approval in Japan
*ex vivo*
	Strimvelis	Severe combined immunodeficiency	Autologous Hematopoietic stem cells retroviral vector GSK3336223	Anaemia, neutropenia, autoimune haemolytic anaemia, autoimune aplastic anaemia, autoimune thrombocytopenia, autoimune thyroiditis, Guillain-Barré syndrome, rhinitis allergic, asthma, dermatites atopic, eczema.	2016 - Approved
	Zalmoxis	Hematopoietic Stem Cell Transplantation	Allogeneic T cells genetically modified with a retroviral vector	Acute and chronic GvHD, febrile neutropenia, haemoglobin decreased, platelet count decreased, hepatic failure, bronchitis and hepatic failure.	2016 - Approved 2019 - Withdrawn
	Kymriah	B-cell precursor acute lymphoblastic leukemia	Autologous T cells - CAR T cell therapy modified with lentiviral vector	Cytokine Release Syndrome. Neurological toxicities. Infections and Febrile Neutropenia. Prolonged Cytopenias. Hypogammaglobulinemia. Other manifestations included seizures, mutism and aphasia.	2017 - Approved
	Yescarta	Refractory large B-cell lymphoma	Autologous T cells - CAR T cell therapy modified by retroviral transduction	Cytokine Release Syndrome. Neurologic toxicities, both including fatal or life-threatening reactions.	2017 - Approved
	Invossa	Knee osteoarthritis	Allogeneic chondrocytes	Under Phase III for safety and efficacy evaluation.	2017 - Phase III Clinical Trial approved in US

^*^ Information obtained from package insert, unless indicated otherwise.

**Table 3 -:** Approved products for gene silencing.

Type	Name	Indication	Method*	Immune-related issues**	Situation
*in vivo*
	Vitravene (fomivirsen)	Cytomegalovirus retinitis	Gene-silencing antisense therapy (ASO - antisense oligonucleotide)	Uveitis, including iritis and vitritis. Conjunctival and retinal inflammation. Anaemia, asthenia, diarrhea, fever, infection, rash, sepsis. More rare: abnormal liver function, allergic reactions, kidney failure, lymphoma like reaction, neuropathy, neutropenia, pancreatitis, thrombocytopenia.	2002 - Withdrawn (EU) 2006 - Withdrawn (US)
	Macugen (pegaptanib)	Neovascular age-related macular degeneration (AMD)	RNA oligonucleotide	Anterior chamber inflammation, punctate keratitis. Rare cases of anaphylaxis/anaphylactoid reactions, including angioedema, have been reported.	2004 - Approved (FDA) 2011 - Withdrawn (EU)
	Kynamro (mipomersen sodium)	Homozygous familial hypercholesterolemia (HoFH)	Gene-silencing antisense therapy (ASO)	Elevation of alanine aminotransferase, Hepatic steatosis, influenza-like illness, pyrexia, arthralgia.	2013 - Approved
	Exondys 51 (eterplisen)	Duchenne muscular distrophy	ASO	Contact dermatitis	2016 - Approved
	Spinraza (nusinersen)	Spinal muscular atrophy (SMA)	Gene-silencing antisense therapy (ASO)	Thrombocytopenia and Coagulation Abnormalities, Renal Toxicity, lower respiratory infection and upper respiratory infection.	2016 - Approved
	Tegsedi (inotersen)	Transthyretin-mediated amyloidosis (hATTR)	ASO	Injection site reactions and fever. Thrombocytopenia, glomerulonephritis, renal toxicity, hepatic dysfunction, strokes. Rarer: antineutrophil cytoplasmic autoantibody (ANCA)-positive systemic vasculitis.	2018 - Approved
	Patisiran (onpattro)	Polyneuropathy caused by hereditary transthyretin-mediated amyloidosis (hATTR)	RNA interference	Upper respiratory tract infections and infusion-related reactions.	2018 - Approved
	Vyondis 53 (golodirsen)	Duchenne muscular distrophy	ASO	Hypersensitivity reaction, rash, pyrexia, pruritus, urticaria, dermatitis, skin exfoliation, nasopharyngitis. Renal toxicity.	2019 - Approved
	Waylivra (volanesorsen)	Lipoprotein lipase deficiency; Hypertriglyceridaemia	ASO	Injection site reactions. Allergic reaction including rash and fever. Thrombocytopenia, renal and hepatic toxicity.	2019 - Approved

^*^ As described in the approval document. ** Information obtained from package insert, unless indicated otherwise.

### The *ex vivo* approved gene therapies

Indicated for treating severe combined immunodeficiency with no suitable stem cell donor, Strimvelis (GSK2696273) is the first *ex vivo* autologous gene therapy approved by EMA in 2016 (EMA, 2016a). The treatment consists of autologous CD34^+^ enriched cells transduced with a retroviral vector encoding the adenosine deaminase (ADA) cDNA sequence. The patient’s hematopoietic stem cells (HSCs) are extracted and purified until only CD34 expressing cells remain. They are then cultured and transduced with a gamma-retrovirus containing the human adenosine deaminase gene. At the end of the process, the cells are re-infused into the patient. The treatment was developed by GlaxoSmithKline (GSK) and had fever as the most common side effect.

Zalmoxis is a gene therapy for hematological malignancies, constituted of T lymphocytes genetically modified to express a shortened variant of the human low-affinity nerve growth factor receptor (LNGFR) and herpes simplex I virus thymidine kinase (HSV-TK Mut2) as a suicide gene (EMA, 2016b). It is an additional therapy for haploidentical hematopoietic stem cell transplantation (HSCT) of adult patients with high-risk hematological malignancies (EMA, 2016b). Infusion of genetically modified donor T cells into HSCT (T cell depletion) transplant recipients can easily restore immunity to protect against infections. Additionally, it may also challenge cancer cells. However, it might target the host’s normal cells, leading to Graft Versus Host Disease (GVHD). In this case, a rescue mechanism using the suicide gene can be triggered using Ganciclovir (Belete, 2021). This drug induces the death of HSV-TK expressing T-cells, controlling GVHD. Zalmoxis was withdrawn after noticing that the product did not affect disease-free survival (EMA, 2019).

Kymriah (tisagenlecleucel) started to be developed by the University of Pennsylvania and was finished by Novartis to treat patients under 25 years old with B-cell acute lymphoblastic leukemia (ALL) or refractory Diffuse large B-cell lymphoma (DLBCL). Kymriah became the first treatment using gene therapy approved in the United States in August 2017 (FDA, 2021a). As with Strimvelis, the treatment is personalized to each patient. Purified T cells extracted from the patient’s blood are modified to make a chimeric cell surface receptor (CAR-T) infused into the patient with the primary goal of targeting the CD19 protein common in B cells (Brandt et al., 2020). The most common side effects of Kymriah are cytokine release syndrome (CRS) and the decrease of platelets, hemoglobin, or white blood cells. Among patients with DLBCL, the side effects might occur in 3 of 10 patients (EMA, 2018a).

Another gene therapy for B cells indicated to lymphoma that failed on conventional treatment is Yescarta (axicabtagene ciloleucel). Kite Pharma submitted it for FDA approval as a treatment for non-Hodgkin lymphoma in 2017. This gene therapy also focuses on genetically modifying immune cells from patients to make them able to act against the tumor cells. The drug mechanism is very similar to Kymriah. The engineered CAR-T cells bind to the tumor B-cells’ CD19 protein, leading to activation of T-cell proliferation and, after, systemic cytokines release such as interleukin (IL)-15 and other chemokines, resulting in cell apoptosis (EMA, 2018b; Kite Pharma, 2017; FDA, 2021b) . Some of the adverse events related to Yescarta involve mainly CRS, correlated with the high levels of IFN-γ, noticed until 12 hours after the infusion. Although the principal symptom is fever, it may include hypoxemia and damage to the heart and kidneys. Neurological events developing up to two weeks following vector administration have also been described (National Library of Medicine [NLM], NCT02348216, 2015; NLM, NCT03391466).

Zynteglo (betibeglogene autotemcel) gene therapy involves modifying the patient’s stem cells with a viral vector made on parts of the human immunodeficiency virus (HIV), modified not to cause AIDS. The virus contains normal copies of the beta-globin coding gene (βA^-T87Q-^globin gene) that are integrated into the cell. These cells are then reinfused into the patient, producing HbAT87Q hemoglobin at levels that considerably reduce the requirement for transfusions (NLM, NCT01745120). First designated as an orphan drug to treat beta-thalassemia, a blood condition that requires monthly blood transfusions in individuals aged 12 and above, in the US in 2015 and for medical purposes in the EU in 2019, Zynteglo has been suspended as a precaution in Europe in February 2021. The allegation was that the viral vector used in this therapy might be responsible for the two cases of acute myeloid leukemia in a clinical trial for treating sickle cell disease. However, the EMA’s human medicines committee (CHMP) has validated the conclusions of a review, published in July 2021, made by the Pharmacovigilance Risk Assessment Committee (PRAC) that found no evidence of vector in Zynteglo caused AML in those patients (PRAC, 2021; EMA, 2021). Although approved in the EU, it is not approved in the US yet.

### The *in vivo* and *in situ* approved gene therapies

Gendicine (recombinant human p53adenovirus [Ad5RSV-p53]) was the first approved gene therapy globally. It was developed by Shenzhen SiBiono GeneTech, in China and approved by the CFDA in 2003 as a treatment for head and neck squamous cell carcinoma with any *KRAS* mutation. The vector enters the tumor cells through receptor-mediated endocytosis and overexpresses p53, stimulating the apoptotic pathways in the cell. The therapeutic product was shown to induce an elevated level of tumor-suppressing gene expression (Zhang et al., 2018).

Oncorine (recombinant Human Adenovirus Type 5 Injection) was also developed to treat head and neck cancer. However, it consists of a modified adenovirus produced by Shanghai Sunway Biotech and was the first oncolytic virus to be approved as a treatment by SFDA in 2005. This treatment was designed to disable a viral defense mechanism that interacts with the p53 gene, commonly dysregulated in cancer cells (Liang, 2018). The mechanism has not been fully understood since the adenovirus used in this therapy targets cells regardless of their p53 status (Garber, 2006). A similar situation, in which viral replication seemed restricted to p53-defective tumor cells, was described by O’Shea et al. (2004). In this case, the authors showed that the absence of E1B-55K results in the induction but not in the activation of p53 during adenoviral replication. Authors suggest that RNA export may be compromised in tumor cells, which would explain the effects of ONYX-015. However, it has been shown to kill the tumor cells preferentially, with better results than chemotherapy only (Jhawar et al., 2017).

Glybera (alipogene tiparvovec) is a gene therapy treatment developed by uniQure biopharma BV for lipoprotein lipase deficiency (LPLD) {OMIM: 238600}. This rare recessive disease causes severe pancreatitis due to mutations in the *LPL* gene. The first approval came from the European Medicines Agency (EMA) in July 2012 (EMA, 2017a,b). The drug, administered as injections in the leg muscles, contains the *LPL* gene encoded by an adeno-associated virus vector and soon has been called the “million-dollar drug” (Bulcha et al., 2021) due to the price of the therapy. Glybera has been used in only one patient, reimbursed by the German government after lots of bureaucracy. This resulted in uniQure withdrawing from the European Union two years after the approval. However, the drug has been supplied at a nominal price for patients approved for treatment until the expiration date on October 25, 2017, by the Italian marketing partner Chiesi Farmaceutici (Senior, 2017).

Developed in Russia in 2010, Neovasculgen (Cambiogenplasmid) is an encoding plasmid DNA for VEGF165, controlled by a cytomegalovirus (CMV) promoter. Composed of a transcription start site, the encoding VEGF165 isoform, a polyadenylation signal, a splicing signal, and an SV40 transcription terminator, Neovasculgen aims to treat atherosclerotic peripheral arterial disease (Bondar et al., 2015). The drug’s intramuscular delivery increases the ankle-brachial index, blood flow velocity, and pain-free walking distance. As a result, it was proposed as a viable treatment option for moderate to severe claudication or limping caused by persistent lower limb ischemia and approved by EMA (Deev et al., 2015).

In 2007, Rexin-G (Mx-dnG1), a tumor-targeted retrovector bearing a cytocidal cyclin G1 construct, received approval from the Philippines FDA to treat all chemotherapy-resistant solid tumors. However, the approval for orphan drug designation came earlier in the United States. The FDA granted Rexin-G market protection for pancreatic cancer in 2003. Later, in 2008, the drug received orphan drug designation for osteosarcoma and soft tissue sarcoma (Orphan Drug Designations, 2008a,b; Chawla et al., 2010; Gordon and Hall, 2010). Still, this drug does not figure as FDA-approved gene therapy.

Imlygic (talimogene laherparepvec), a genetically engineered herpes virus, was developed to be delivered by injection directly in inoperable melanoma. The virus is two genes absent and is modified to have the human *​​GM-CSF* gene. These modifications stimulate the immune system against the patient’s tumor cells. The drug developed by Amgen was the first oncolytic immunotherapy approved by the FDA in October 2015 (FDA, 2015a,b).

Spark Therapeutics has developed and commercialized Voretigene Neparvovec-rzyl (AAV2-hRPE65v2), called Luxturna. It is the first gene therapy for inherited conditions authorized by the FDA in the United States on December 19, 2017, and by EMA on November 23, 2018 (FDA, 2018; EMA, 2018). Luxturna is an orphan medication used intraocularly to treat hereditary retinal degeneration caused by biallelic *RPE65* mutations (Ramlogan-Steel et al., 2019). The clinical phenotypes of Leber congenital amaurosis type 2 (LCA2) {OMIM:204100} and retinitis pigmentosa type 20 (RP20) {OMIM: 613794} are caused by this kind of inherited retinal dystrophies (IRD). Retinitis pigmentosa (RP) is the most prevalent type of IRD. Both LCA2 and RP20 have an autosomal recessive inheritance pattern. The isomerase deficiency in the *RPE65* gene causes retinal pigment epithelium cells to lose their capacity to respond to light (Chung et al., 2018). The therapeutic virus contains copies of the *RPE65* normal gene. The injection of Luxturna into the eye leads to the virus infection of retinal cells, allowing them to produce the missing enzyme and reducing the disease’s development. The adeno-associated virus employed in this treatment does not cause any illness in humans (EMA, 2018).

Zolgensma (onasemnogene abeparvovec-xioi) is one of the most recent gene therapies approved by the FDA (May 2019). It is the first gene therapy approved to treat children under two years of age, carriers of spinal muscular atrophy (SMA) {OMIM: 253550}. This is one of the most severe forms of the disease and is a prominent hereditary cause of infant death. A mutation in the *SMN1* gene is the cause of SMA as the SMN protein encoded by this gene is essential for the maintenance and function of specialized nerve cells known as motor neurons. These cells, present in the brain and spinal cord, control muscle movements. In children, the signs and symptoms of the disease may appear at birth or by the age of six months (FDA, 2021c). The therapy consists of a non-replicating recombinant AAV9 with a functional copy of the human *SMN1* gene controlled by the CMV enhancer/chicken-actin-hybrid promoter (CB) to express *SMN1* in SMA patients’ motor neurons. The AAV9 capsid’s ability to penetrate the blood-brain barrier allows effective CNS delivery by intravenous injection. Additionally, the AAV ITR is modified to create a self-complementary DNA molecule that makes a double-stranded transgene that improves active transcription (Waldrop and Kolb, 2019).

Delytact (teserpaturev/G47∆) is the gene therapy made by Daiichi Sankyo company conditionally approved by Japan’s Ministry of Health, Labour and Welfare (MHLW) to treat malignant glioma in June 2021. The drug is based on genetically modified oncolytic herpes simplex virus (oHSV) type 1. The drug has a triple mutation on the viral genome, implying selective replication in cancer cells. G207 was the first oHSV approved in gene therapies (Herbring et al., 2016). The third generation of this oHSV (G47∆) results from the deletion of infected cell proteins (ICP)47 that places the late Us11 gene, a PKR inhibitor, under the control of the immediate-early ICP47 promoter. These changes block the protein shut-off (Jahan et al., 2021). Once MHC-I expression is absent due to the ICP47 presence in HSV-infected cells, the human lymphocytes more efficiently recognize the antigens from the tumor and the virus in cancer cells when the ICP47 is deleted on the oncolytic vector (Jahan *et al.,* 2021; Zeng et al., 2021). The conditional approval was received considering the results of the phase II trial (UMIN000015995, 2014) (Daiichi Press Release, 2021).

## The immune response

**Figure 4 -:**
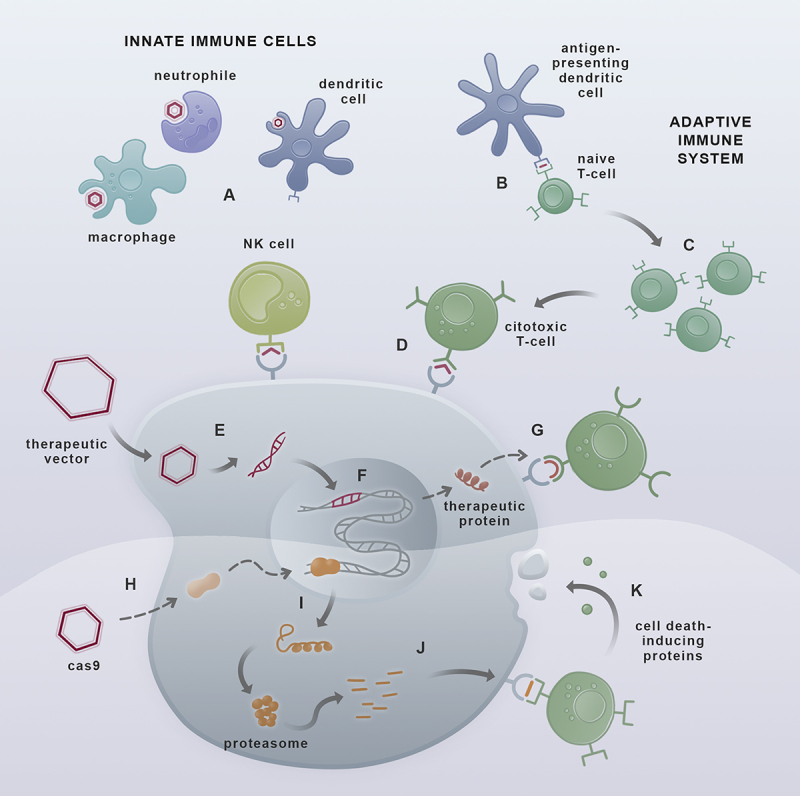
Immune barriers to gene therapy and gene editing. Under infection, the primary defenses are the cells from the innate immune system. Here the viral content can be recognized and destroyed by the different phagocytic cells or recruiting other cells through specific cytokines such as the dendritic cells or natural killer cells (NK) that destroy infected cells upon specific receptor interactions (**A**). The second layer of response can be triggered by antigen-presenting cells that connect the innate and the adaptive immune systems (**B**). This contact results in the proliferation of naive T cells (**C**) that respond against the antigen through effector T cells (**D**). When the vector evades the innate immune system, the response may occur upon the recognition of parts of the vector (**E**) or, after the integration of the transgene into the host genome (**F**) under the recognition of the transgene product as non-self (**G**). The intensity of this response may depend on the partial existence of the gene product to be inserted. Finally, the gene-editing approach (**H**) presents an additional immune target: the editing protein itself. After promoting the gene edition, the protein follows the degradation pathway (**I**), resulting in small foreign peptides (**J**) that might be presented to cytotoxic T cells. In any case, CD8+ activation leads to the production of proinflammatory cytokines, resulting in cell death (**K**).

As shown above, different strategies are employed in approved gene therapy protocols. However, regardless of the vector or the targeting approach, the immune response against the vector and the transgene are significant challenges that gene therapy products may face. To be effective *in vivo*, gene therapy treatments must often overcome three significant immunological barriers (Wagner et al., 2021), as resumed above and in Figure 4:

- Avoid antibody neutralization of the delivery system;
- Avoid response against the vector or its content after delivery;
- Avoid immune response against the product of the corrected gene.

All these issues may be circumvented with different strategies that depend on the type of vector and how they interact with the immune system.

### Immune response against the vectors

Non-viral vectors are safer and easier to build but pose significant cell targeting and transfection efficiency challenges. Generally, strategies to reduce liposome and nanoemulsion opsonization use polyethylene glycol (PEG) (Shi et al., 2021). Even naked DNA strategies may present immune activation, either against DNA itself or contamination from bacterial products. Although seldom remembered, bacteria are essential players in gene therapy, as most constructs and plasmids are grown inside bacterial cells. Adequate purification procedures, however, make their participation in immune response unlikely.

Viruses have a natural ability to manipulate foreign genetic content into the host genome and therefore are preferred in terms of efficacy of gene transduction. On the other hand, viral vectors are often related to immune responses. When a pathogen invades the organism, an innate response is triggered to prevent infection. Cytokine production and the recruitment of nonspecific inflammatory cells, such as macrophages, NK cells, and others, are activated by toll-like receptors recognizing pathogenic peptides in the viral capsid.

*Adenoviral vectors*

Adenoviral vectors are non-enveloped double-stranded DNA vectors with a capacity of packing around 35kb (Talmadge and Cowan, 2020). The AdV vectors may be divided into two regions: early (E) - comprehending E1, E2, E3, and E4 regions, and late (L) - L1, L2, L3, L4, and L5 regions, each one named according to the time of their expression during the virus replication. Adenoviruses are common respiratory viruses that most people have contact with. Zsengellér and colleagues evaluate the central role of the alveolar macrophages during an adenovirus respiratory tract infection. They have shown that 30 minutes after the infection, alveolar macrophages started to express TNF-α and IL-6 in murine models (Zsengellér *et al.,* 2000). In primates, this response may take a little more time, as the systemic production of IL-6 and macrophage activation occurs around 2 hours after the administration in monkeys (Schnell et al., 2001).

The first generation of AdV vectors was known for triggering the immune system. Even the absence of the E1 region, lowering the chances of expression of viral genes, did not completely diminish the viral replication capability. In terms of the immune system, this is enough to start the activation of cytotoxic T cells (CTLs) (Lusky et al., 1998). This strong response has resulted in acute inflammation for several patients and death for a participant of a clinical trial (NLM, NCT00004386, 1999) that used an adenoviral vector for ornithine transcarbamylase deficiency (OTC) {OMIM: 311250} (Lehrman, 1999; Wilson, 2009).

The patient received an administration of 6x10^11^ particles/kg of an AdV type 5 preparation on the right hepatic artery. The first adverse reactions were noticed 18 hours after the administration and consisted of jaundice and altered mental status. Systemic inflammatory response syndrome and disseminated blood clots were reported in the subsequent hours. The death occurred 98 hours after the administration from multiple organ system failures. In the post-mortem analysis, it was possible to identify high levels of IL6, IL10, and TNF-a, in comparison with other participants in the study (Raper *et al.,* 2003).

Although the last generation of adenoviral vectors - characterized by the deletion of all viral genes from the vector - shows a favorable adaptive response, it still induces an innate response (Rogers et al., 2011). The activation of innate response is dose-dependent and limited to a certain threshold (Ronzitti et al., 2020). This response can be used for therapeutic purposes. One of the applications is DNX-2401 (Delta-24-RGD; tasadenoturev), a tumor-selective replication-competent oncolytic adenovirus designed as a monotherapy to treat long-term CRs in glioblastoma (Lang *et al.,* 2018). Although the mechanisms are still not fully understood, the therapy has shown anti-glioma efficacy, with tumor regression being observed several months after the administration without viral replication detection, suggesting that the effect is likely due to immune response. (Ferrera-Sal et al., 2021).

*Adeno-associated viral vectors*

First discovered from laboratory AdV preparations (Atchison et al., 1965), adeno-associated vectors are small non-enveloped viruses with a single strand DNA genome (4.7 Kb) contained in an icosahedral capsid. It was found not to be pathogenic in humans (Rogers et al., 2011) many years later than its first discovery in human tissues (Blacklow et al., 1967). All the research on adeno-associated virus (AAV) to characterize and understand its composition, replication, and transcription process and assembly made it possible for scientists to clone AAV into plasmids.

The AAV genome comprises two genes, rep and cap, flanked by two palindromic inverted terminal repeats (ITR). Rep codes for proteins involved in viral DNA replication, AAV genome packing, and viral genome integration into the host DNA (Wang et al., 2019). Cap genes encode the capsid proteins and can be switched between serotypes (pseudotyping). Additionally, it produces two accessories proteins: AAP (assembly activating protein) and MAAP (membrane assembly activating protein) through alternative open reading frames (Sonntag *et al.,* 2010; Ogden et al., 2019). AAV stays latent in integrated or non-integrated forms after infection until a helper virus offers the required functions for its replication (Ronzitti et al., 2020).

AAV seems not to induce the IL-8, IP-10, and RANTES chemokines expression, in contrast to adenoviral vectors (Zaiss et al., 2002), which do so in a dose-dependent manner. Due to this lack of pathogenicity, AAV became the preferred vector for many applications (Kuzmin et al., 2021) and is the base for approved gene therapy drugs cited before: Glybera, Luxturna, and Zolgensma for lipoprotein lipase deficiency (LPLD), inherited retinal disease (IRD), and spinal muscular atrophy (SMA), respectively.

AAV can elicit a cell-mediated action by the immune system even without triggering any innate response. Zhang et al. (2000) showed this response occurring *in vitro* when immature dendritic cells from femurs and tibias of 8- to 10-week-old female C57BL/6 mice incorporate the vector; and *in vivo* after adoptive transfer. This characteristic can also be used for oncolytic gene therapy. For example, Liu et al. (2001) showed that AAV-mediated transduction of dendritic cells led to increased cytokine production. Using the system to deliver the HPV-16 E6 antigen gene into the cells, they could induce a class I (MHC-I)-restricted cervical cancer cell killing activity.

Severe adverse events caused by immune responses against AAV have recently been described. Three young males died after receiving the highest dose (3.5×1014 vg/kg) of AAV to treat X-linked Myotubular Myopathy (NLM, NCT03199469, 2018). Later, another boy died after receiving the lowest dose (1.3×10^14^ vg/kg) in the same study. The first side effects were liver dysfunction, followed by progressive cholestatic hepatitis and liver failure. In the mini-dystrophin gene therapy study from Pfizer (PF-06939926), the death of a patient was also related to immune response due to AAV high dose (Agarwal, 2020). These cases show the importance of considering immune responses more deeply in AAV clinical trials.

However, as in the AdV cases, this response may favor gene therapy. This is the case of oncolytic vectors such as Imlygic, which stimulates the immune system against the patient’s tumor cells using an attenuated HSV-1 that enhances the preferential tumor-killing property of the virus.

*Lentiviruses*

Lentiviruses are a subtype of retrovirus capable of infecting nondividing and actively dividing cells. They are composed of single-strand RNA converted to double-strand DNA during their replication process. Retroviruses’ general gene composition, also present in lentiviruses, is gag (the precursor to structural proteins), pro (protease enzymes), pol (integrase and reverse transcriptase precursors), and env (precursor to envelope glycoproteins) (Krebs et al., 2021). The main issues linked to these vectors are insertional mutagenesis and genotoxicity (David and Doherty, 2017; Morgan et al., 2021). Indeed, in 2002, in a trial for X-linked severe combined immunodeficiency, four out of ten patients developed leukemia presumably associated with vector integration (Hacein-Bey-Abina et al., 2003).

This vector type, typically derived from HIV1, has primarily replaced retrovirus due to safety concerns and is involved in 10% of all gene therapy clinical trials worldwide (Ginn et al., 2013), both *in vivo* and *ex vivo*. Most commonly, they are derived from primate lentiviruses Human Immunodeficiency Virus Type 1 (HIV-1) (Reiser et al., 1996) and Type 2 (HIV-2) (Arya et al., 1998) and Simian Immunodeficiency Virus (SIV) or non-primate lentiviruses (Schnell et al., 2000), Feline Immunodeficiency Virus (FIV) (Poeschla et al., 1998) and Equine infectious anemia virus (EIAV) (Olsen, 1998). 

Brown et al. (2007a) were the first to demonstrate innate response in mice following LV delivery. Overall, the multiple interactions of LVs with the innate immune system occur depending on the LV dose, pseudotype, method of production, model strain, or recipient species remains (Annoni et al., 2019). The primary responses are mediated by IFN-α,β production; pDC and cDC activation; and TLR-7 signaling (Nayak and Herzog, 2009).

*In vivo* therapies are in the pre-clinical phase (Palfi et al., 2018; Link et al., 2020), but the immune response can limit effectiveness and safety. The administration of the vector leads to a humoral and cell-mediated response against the LV capsid that may culminate in the inactivation of the vector, abrogating transduction, or eliminating transduced cells while the system is still exposed to the LV-derived antigens. However, vector re-administration and detailed characterization of anti-LV immune responses after systemic delivery still need to be investigated in animal models. Moreover, allogeneic immune responses can still occur against LVs produced by human-derived cells (Annoni et al., 2019). A crucial point to be observed is the known ability of the LV vectors to transduce APC cells. Transduction of Kupfer cells (liver), macrophages, B cells, and dendritic cells in the spleen has been demonstrated by Van den Driesschee et al. (2002). 

The parental HIV-1 elicits cell- and antibody-mediated responses in humans. Their immunogenicity indicates that LVs can activate innate and adaptive immunity (Follenzi et al., 2007). One limiting factor on gene therapies that use HIV as a vector for application in patients is the pre-existing immunity to the wild-type virus (Annoni et al., 2013). However, the persistence of the LV-modified T cells has been demonstrated in a clinical trial (NLM, NCT00295477) that administered autologous T cells in HIV-infected patients. This result suggests that pre-existing anti-HIV immunity is not enough to affect the efficiency of gene therapy. The most probable explanation is that even after receiving multiple infusions, the modified T cells did not carry over LV- or VSV.G-derived antigens (Annoni *et al.,* 2019).

*Ex vivo* therapies using hematopoietic stem cells and progenitors (Tucci et al., 2021) or T cells (Ribeil et al., 2017) are under clinical trials (NLM, NCT01852071; NLM, NCT01515462, 2012) and have shown satisfactory results in the use of HSPCs and LVs. Although recipients are not directly exposed to LVs, as in the i*n vivo* gene therapy, there is some risk of an immune response induced by the carry-over of antigens derived from the vector by the infused cells.

Still, the main issue about LV triggering immune responses is not related to the vector itself but the reactions against the transgene product. The intensity of this response may lead to a clearance of all cells that express the transgene proteins, as explained in the next topic.

### Immune response against the transgene

The risk of response against the transgene is dependent on factors such as the type and route of the vector administration and the target tissue. Also, it depends on the host’s characteristics, such as disease-specific tissue inflammation and the amount of pre-existing gene product (Shirley et al., 2020). It has been shown that the innate immune system can regulate the transgene expression through the IFNγ and TNFα cytokines, inhibiting transgene expression (Sung et al., 2001).

The response against transgene products might happen with different intensities, depending on host genomic alteration, a process also observed in enzyme replacement therapy (ERT). Knowing if the patient expresses a truncated form of the protein or does not express it is relevant to assessing the intensity of an immune response. In the first case, the immune response against the protein is attenuated since it would be dependent on the neo-antigens derived from the therapeutic protein. In the second case, however, the absence of the natural protein might result in a more intense immune response. In a nonrandomized study (NLM, NCT00882921) for evaluation of the idursulfase long-term ERT for mucopolysaccharidosis II patients, it was found that 50% of the patients presented idursulfase-specific IgG antibodies (Giugliani et al., 2017). 

While a humoral response is expected on ERTs (Lenders and Brand, 2018), in gene therapy, it is most likely developing a cellular response against the transgene product. In phase I/II gene therapy study, patients with Duchenne’s muscular dystrophy had a mini-dystrophin gene transferred by AAV (Mendell et al., 2010). The result was a poor protein expression caused by possible pre-existing T cell immunity because of occasional endogenous dystrophin expression in revertant fibers. Again, this issue is not exclusive to gene therapy. Previously, Tremblay et al. (1993) showed the induction of immune response against the dystrophin after transplantation of myoblasts into the cardiac tissue of Duchenne patients.

Another issue not directly related to the transgene but that may affect it is the innate response against the promoters-generated RNA from transgenic AAV cassette that results in opposite complementary transcripts, triggering an innate response shown by Shao et al. (2018). It was demonstrated by measuring the levels of the transgene, which was increased when a plasmid with the 3′-ITR deletion and decreased with the insertion of a reversed polyA sequence between 5′-ITR and the start codon.

### Immune response against the gene-editing proteins

Gene editing presents an additional target to the immune system: the reaction against the proteins used for double-strand DNA break. Although a lot has been done to understand the mechanisms of B-cell response against the vectors that deliver CRISPR/Cas9 into the cells, the T-cell response has been just recently shown.

Wang et al. (2015) found T-cell response against Cas9 proteins in immunocompetent mice. An adenovirus vector delivered a *Streptococcus pyogenes*-derived Cas9 system targeting the *Pten* gene, a frequently mutated gene in patients with sporadic cancer and involved in nonalcoholic steatohepatitis (NASH). The authors showed that hepatocytes were lost under humoral and cellular immune response against the AdV vectors between two weeks and four months after the injection. But they also noted an immune response against the Cas9 protein, detected through an ELISA assay for SpCas9 antibodies. In addition, a robust IgG1 antibody formation against Cas9 fourteen days after the administration of the adenovirus was reported.

Chew et al. (2016) and Chew (2018) have shown the same response when using AAV or electroporation to overexpress a transgene in the same type of animal. In 2016, the authors tested the functionality of AAV-Cas9-gRNA targeting *Mstn* (AAV9-Cas9-gRNA^M3+M4^) by intraperitoneal injection on neonatal mice. The ELISA assay has confirmed a Cas9-specific humoral immune response, and Cas9 peptides were mapped using serum from the animals with M13 phage libraries covered with the Cas9 transgene. The T-cell response has also been found in studies that overexpressed SpCas9 in tumors transplanted into immunocompetent mice (Ajina et al., 2019). Moreover, Li et al. (2020) demonstrated that the immunizations with SaCas9 in mice a week before the delivery of AVV-liver therapy decreased the long-term survival of the *in vivo* edited hepatocytes. This suggests an immune response against treatments mediated by Cas9 proteins due to memory acquired from previous infections.

Crudele and Chamberlain (2018) shed light on a pre-existent response against the most common human pathogens, *Staphylococcus aureus* and *Streptococcus pyogenes*, known for causing MRSA and strep throat from whom Cas9 protein is derived. Charlesworth et al. (2019) showed that our immune system could recognize Cas9 peptides as non-self, and the prevalence of anti-Cas9 response in healthy human adults is 79% anti-Cas9 IgG for SaCas9 and 67% for SpCas9. Simhadri et al. (2018), on the other hand, found rates around 10% and 2.5% for anti-SaCas9 e anti-SpCas9 in samples from the US. 

Works from Wagner et al. (2019) and Ferdosi et al. (2019) have found similar results, pointing out that 85% and 5% of the blood donors have anti-SpCas9 antibodies and anti-SpCas9 T cells. While Charlesworth and Wagner’s works have shown such a response using the entire recombinant protein through ELISPOT and flow cytometry, Ferdosi *et al*. (2019) used a different approach. Using *in silico* tools, they selected and built a pool of 38 peptides to test using HLA-A*02:01 pentamers associating ELISPOT and flow cytometry, reporting 83% (n =12) of the sample with IFN-γ+ response. Stadtmauer et al. (2020) used the same pool of peptides and suggested that 66% of the sample (n=3) are responsive to SpCas9. In addition, they reported the first human clinical trial designed to test the safety and feasibility of CRISPR-Cas9 editing of T cell receptors. 

## Avoiding the immune system

As interaction with the immune system may hamper gene therapy results, there is a need for countermeasures. Sack and Herzog (2009) divide the alternatives to circumvent the immune response into two categories

- Methods that hide the vector or/and the transgene product from the immune system;
- Methods that hide the immune system from the vector/transgene product.

**Figure 5 -:**
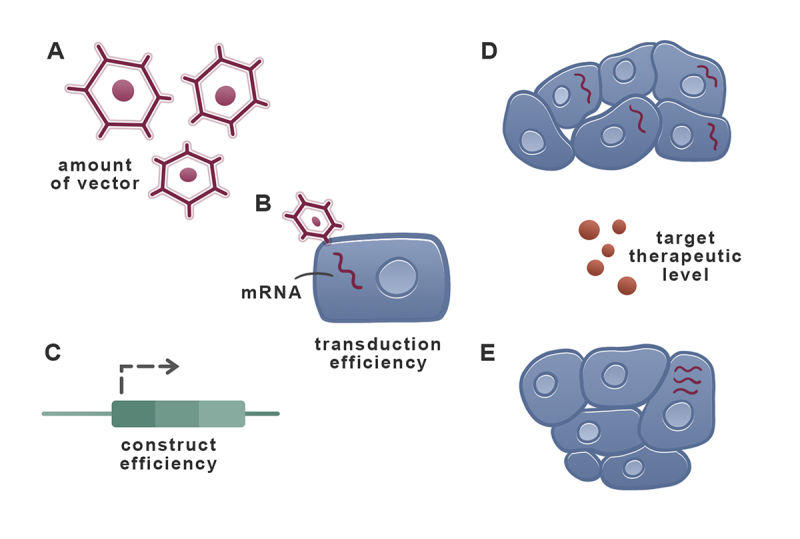
Different strategies that may be used independently or combined to achieve target therapeutic levels of the transgene, in this case a secreted protein. Ideally, the amount of vector can be controlled in order to decrease the immune response (A). This can be compensated by vectors with higher transduction efficiency (B) and/or constructs with higher transgene expression (C). The desired outcome is a large number of transduced cells expressing the transgene in physiological levels (D) as opposed to a few high-expressing cells that may be more easily detected by an immune response against the therapeutic protein (E).

In the first scenario, it is possible to decrease the vector dose through capsid or transgene modifications or to use hydrodynamic injections. It is also possible to deliver the therapy only on immune-privileged sites such as the eyes, brain, knees, or liver. More sophisticated strategies include preventing the APC expression through tissue-specific promoters or miRNA targeting. Hiding the immune system from vectors can be achieved by suppression or modulation. One of the most common suppression mechanisms is to block cell division. Another is the depletion of specific cell types with antibodies. In immune modulation, it is possible to induce or adoptively transfer regulatory T cells (Tregs) or block the co-stimulation. In any of these processes, a balanced strategy to keep therapeutic levels of target proteins is desired (Figure 5).

### Hiding the vector from the immune system

From the vector to the transgene, lots of work has been done on avoiding immune response and improving the outcomes of gene therapy techniques. Lowering adenoviral vector doses was one of the first measures. This strategy is effective in hepatic gene transfer, including developing immune tolerance to coagulation factor IX (see Mingozzi and High, 2007). However, low doses of AdV may be quickly neutralized by the immune system in such a way that must be compensated by an increase of efficiency for the gene therapy to occur correctly.

The most studied methods to evade the immune system have been the modifications in the viral capsid since the response, toxicity, and clearance result from the interaction between the viral capsid and the host cells (Ahi et al., 2011). With this in mind, covalent modifications have been developed to change the immunodominant epitopes and the capsid components needed for this interaction, as in studies using PEG.

PEG is an FDA-approved substance that allows covalent coupling of proteins, modifying major capsid proteins, fibers, hexons, and pentons. Croyle et al. (2002) demonstrated lower levels of immune response specific to AdV and increased transgene expression *in vivo* using PEGylation of E1 depleted AdV vector in liver cells of murine models.

Several capsid modifications to insert specific sequences that improve the binding to adapter molecules have also been reported. For example, human adenovirus serotype 5 (HAd5) infection occurs through interactions between the AdV fiber knob and some surface cell receptors such as Coxsackievirus-adenovirus receptor (CAR) (Excoffon, 2020), heparan sulfate glycosaminoglycan (Smith et al., 2003; Mitra et al., 2021), or sialic acid saccharide (Arnberg et al., 2000). Fiber knob modifications consist of a knob-specific neutralizing antibody complex that retargets the Adv to another receptor (Bradley et al., 2012). This new chimeric receptor confers to the vector the ability to decrease its immunogenicity.

Capsid modifications are also used for AAV through rational design (Bartel et al., 2011). Lochrie et al. (2006) demonstrated that mapping the immunodominant epitopes of AAV-2 and their mutagenesis were enough to reduce the neutralization by the murine and human immune cells. The modifications were performed in 64 positions (especially on glycines and alanines) on the external surface of the AAV-2. While the reduced neutralization by the monoclonal antibody (A20) on murine accounted for more minor modifications, for human sera or IVIG (purified human IgG), the neutralization was increased when more mutations were combined.

Capsid modifications are also used for AAV. Rational design of the AAV capsid is one of the approaches (Bartel et al., 2011), and the work from Lochrie et al. (2006) have demonstrated that mapping of the immunodominant epitopes of AAV-2 and their mutagenesis were enough to reduce the neutralization by the murine and human immune cells. The modifications were performed in 64 positions (especially on glycines and alanines) on the external surface of the AAV-2. While the reduced neutralization by the monoclonal antibody (A20) on murine accounted for more minor modifications, for human sera or IVIG (purified human IgG), the neutralization was increased when more mutations were combined.

Brown et al. (2007b) have found an interesting solution to avoid an immune response against the transgene and the LVs. They took advantage of the miRNA regulation system, incorporating copies of a mirT targeting miRNA highly expressed in hematopoietic cells with hepatocyte-specific promoters. The strategy prevented the off-target expression of the transgene in hematopoietic-derived cells and resulted in a tissue-specific therapy.

Milani et al. (2017) showed that modifying LVs producing cells to inhibit the MHC I complex expression reduces the immunological response against the vectors. This is due to the attachment of cell surface proteins in the LV capsid. Another solution for response against LVs is the development of integration-defective lentiviral vectors (IDLVs) studied as a vaccine platform for antigen delivery. IDLVs are non-replicating, non-integrating vectors that incorporate a mutated integrase protein, preventing genome integration (Gallinaro et al., 2018). Whereas it also prevents insertional mutagenesis, the vector’s transduction efficiency *in vitro* and *in vivo* remains high, as shown by Wanisch and Yáñez-Muñoz (2009). Mátrai et al. (2011) have also demonstrated the benefits of the induction of active tolerance to the transgene and transgenic-specific Tregs in hepatocyte-targeted IDLV gene transfer due to their low but long-lasting transgene expression.

While a complete non-immunogenic Cas9 protein seems to be a distant reality for gene editing, engineered selective mutations based on the immunogenic peptide studies may be an alternative, as shown by Ferdosi et al. (2019). They show that silencing one peptide for HLA-A:02:01 was enough to diminish the Ca9 immunogenicity for the other three HLA-A alleles. Considering that human populations have around 19,000 HLA alleles (Robinson et al., 2015), the above results are promising, as they show a common approach capable of considering the particularities within populations. 

Although HLA is the main responsible for defining which peptide will be presented to lymphocytes (Gfeller and Bassani-Sternberg, 2018), different sizes of peptides must be considered to properly represent the immunogenic peptides and the modification of the Cas9 protein. On the other hand, developing new types of Cas9 may be a much more feasible attempt. For instance, the miniature CRISPR-Cas system (Harrington et al., 2018; Xu et al., 2021) is now in its early steps for gene editing. The authors showed that the system, with half the size of a Cas9 and Cas12, is efficient and very specific for gene activation, and it allows not only genome editing but also base editing. Intuitively, one may think that half of the sequence, half of the problems in the immune system and adaptive response. However, nothing has been shown about the capabilities of this Cas9 to stay out of the immune surveillance sight.

As a last resource, in many clinical trials, patients are methodically chosen. Patients with low titers of antibodies against the viral vector selected are less likely to present an immune response against the vector. In addition, patients with residual protein levels should offer a less aggressive immune response against the therapeutic product. For example, the developing therapies for hemophilia A and B, until 2016, recruited for their clinical trial only patients that did not present any inhibitory antibody against the protein administered in replacement therapies (Dolgin, 2016).

### Hiding the immune system from the vector

Many drugs used for organ transplantation and autoimmune diseases may be used in gene therapy to modulate the immune response, avoid cell elimination, and promote tolerance. One evidence of such an approach is the use of rapamycin and IL-10 in gene therapy studies of canines with hemophilia (Nayak and Herzog, 2009). Another uses cyclosporine and anti-thymocyte globulin in dogs with Duchenne muscular dystrophy treated with AAV vectors (Wang et al., 2007).

In humans, literature has shown that immune responses may be attenuated after ocular gene therapy using steroid drugs (Chan *et al.,* 2021). However, this strategy is not failure-proof. In hemophilia, the administration of oral immunosuppressors (IS) has failed to prevent immune response against the AAV vector proteins, limiting the efficiency of the therapy in patients (Mingozzi and High, 2013).

Samelson-Jones et al. (2020) showed that the moment of T cell-directed IS administration is crucial in determining transgene-product tolerance. Through a nonhuman primate model and using rabbit thymocyte-globulin (ATG), they evaluated the intensity of T cell response against AAV-mediated transfer of human factor IX (FIX). The results showed that anti-FIX antibody production occurred when the ATG was administered concomitantly with the AAV but was not found when the ATG was delayed five weeks after vector administration.

## Conclusions

Gene therapy has come a long way from its first days and represents one of the major advances in genetic disease treatment. During its development, more than one well-known or recently discovered biotechnological tool has been studied as a tool for gene therapy: recombinant DNA, RNAi, gene delivery, and CRISPR. The possibility of curing genetic diseases and improving the lifespan of cancer patients are part of gene therapy’s promises. However, even being an exciting field, safety always remains a point of concern, with the immune response as an obstacle that must be faced to guarantee the benefits of gene therapy.

Viral vectors were always a point of interest given their gene delivery ability, especially how some of them can circumvent the immune system. Although not discussed in this review, non-viral vectors are a promising tool for gene therapy. However, much improvement is needed, in particular related to transfection efficiency. Despite the variety of non-viral vectors developed in recent years, many still have problems related to stability in a physiological environment, uptake, and endosome evasion, as pointed out by Thapa and Narain (2016).

Studies focused on the transgene also show the importance of improving the understanding of the immune response and immune tolerance mechanisms. Santos et al. (2017), highlighted the use of different animal models that suggested Syrian hamsters as models for understanding oncolytic adenoviruses mechanisms while using mice to obtain detailed immunological analyses. The use of gene-editing technologies poses an additional challenge due to the immune response against the nuclease. In this sense, the development and use of bioinformatics tools to predict and redesign immunogenic epitopes may be helpful.

Finally, combining different strategies, such as patient screening, intelligent vector design, forms of immunosuppression, or inducing tolerance, seems to be the right path for safer and efficient treatment. As new strategies progress, gene therapy makes its way into clinical practice. But the immune system, designed to protect us from foreign nucleic acid molecules, has a hard time understanding when these are used for our benefit.
